# (*E*)-1-(3-Bromo­phen­yl)-3-(4-ethoxy­phen­yl)prop-2-en-1-one

**DOI:** 10.1107/S1600536808018850

**Published:** 2008-06-28

**Authors:** Hoong-Kun Fun, Suchada Chantrapromma, P. S. Patil, S. M. Dharmaprakash

**Affiliations:** a X-ray Crystallography Unit, School of Physics, Universiti Sains Malaysia, 11800 USM, Penang, Malaysia; b Crystal Materials Research Unit, Department of Chemistry, Faculty of Science, Prince of Songkla University, Hat-Yai, Songkhla 90112, Thailand; c Department of Studies in Physics, Mangalore University, Mangalagangotri, Mangalore 574 199, India

## Abstract

The title compound, C_17_H_15_BrO_2_, adopts an *E* configuration. The dihedral angle between the two benzene rings is 10.09 (11)°. The enone plane makes dihedral angles of 12.05 (11) and 9.87 (11)°, respectively, with the bromo­phenyl and ethoxy­phenyl rings. The eth­oxy group is nearly coplanar with the attached benzene ring. In the crystal structure, the mol­ecules are linked by C—H⋯O hydrogen bonds, forming a zigzag ribbon-like structure along the *b*-axis direction.

## Related literature

For bond-length data, see: Allen *et al.* (1987 ▶). For related structures, see: Patil, Fun *et al.* (2007 ▶); Patil, Ng *et al.* (2007 ▶); Sathiya Moorthi *et al.* (2005*a* ▶,*b* ▶). For background to chalcones, see: Chopra *et al.* (2007 ▶); DiCesare *et al.* (2000 ▶); Gu *et al.* (2008*a* ▶,*b* ▶); Jiang *et al.* (1994 ▶); Lokaj *et al.* (2001 ▶); Low *et al.* (2002 ▶); Nel *et al.* (1998 ▶); Patil & Dharmaprakash (2007 ▶); Patil *et al.* (2006 ▶); Schmalle *et al.* (1990 ▶); Wang *et al.* (2004 ▶).
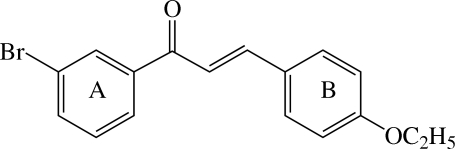


## Experimental

### 

#### Crystal data

C_17_H_15_BrO_2_
*M* *_r_* = 331.19
Monoclinic, 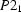

*a* = 4.0516 (1) Å
*b* = 9.6501 (2) Å
*c* = 17.9120 (4) Å
β = 92.396 (1)°
*V* = 699.72 (3) Å^3^
*Z* = 2
Mo *K*α radiation
μ = 2.94 mm^−1^
*T* = 100.0 (1) K
0.53 × 0.31 × 0.17 mm

#### Data collection

Bruker SMART APEXII CCD area-detector diffractometer
Absorption correction: multi-scan (*SADABS*; Bruker, 2005 ▶) *T*_min_ = 0.305, *T*_max_ = 0.641 (expected range = 0.289–0.607)
14837 measured reflections
5989 independent reflections
4682 reflections with *I* > 2σ(*I*)
*R*_int_ = 0.033

#### Refinement

*R*[*F* ^2^ > 2σ(*F* ^2^)] = 0.034
*wR*(*F* ^2^) = 0.093
*S* = 1.04
5989 reflections
182 parameters
1 restraint
H-atom parameters constrained
Δρ_max_ = 0.69 e Å^−3^
Δρ_min_ = −0.65 e Å^−3^
Absolute structure: Flack (1983 ▶), 2764 Friedel pairs
Flack parameter: 0.021 (8)

### 

Data collection: *APEX2* (Bruker, 2005 ▶); cell refinement: *APEX2*; data reduction: *SAINT* (Bruker, 2005 ▶); program(s) used to solve structure: *SHELXTL* (Sheldrick, 2008 ▶); program(s) used to refine structure: *SHELXTL*; molecular graphics: *SHELXTL*; software used to prepare material for publication: *SHELXTL* and *PLATON* (Spek, 2003 ▶).

## Supplementary Material

Crystal structure: contains datablocks global, I. DOI: 10.1107/S1600536808018850/ci2619sup1.cif

Structure factors: contains datablocks I. DOI: 10.1107/S1600536808018850/ci2619Isup2.hkl

Additional supplementary materials:  crystallographic information; 3D view; checkCIF report

## Figures and Tables

**Table 1:** Hydrogen-bond geometry (Å, °)

*D*—H⋯*A*	*D*—H	H⋯*A*	*D*⋯*A*	*D*—H⋯*A*
C9—H9*A*⋯O1	0.93	2.36	2.746 (3)	105
C16—H16*B*⋯O1^i^	0.97	2.49	3.400 (3)	157

Symmetry code: (i) 
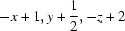
.

**Fig. 1.**
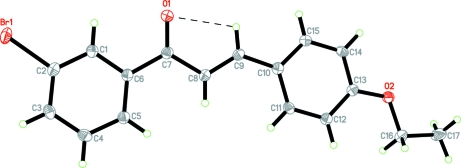
The molecular structure of the title compound, showing 50% probability displacement ellipsoids and the atom-numbering scheme. The dashed line represent a C—H···O interaction.

**Fig. 2.**
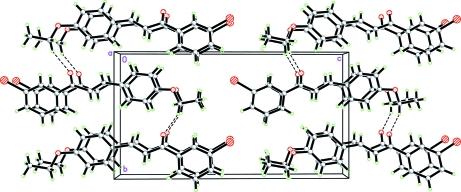
The crystal packing of the title compound, viewed along the a axis. Hydrogen bonds are shown as dashed lines.

**Fig. 3.**
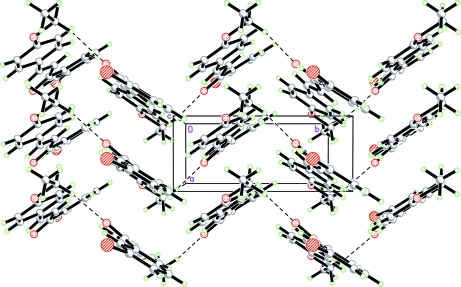
The crystal packing of the title compound, showing zigzag ribbon-like structure running along the b axis. Hydrogen bonds are shown as dashed lines.

## supplementary crystallographic information

### Comment

Extensive research in recent years suggests organic materials to be the ideal candidates for tailoring the material properties. As an interesting type of organic materials, chalcone and its derivatives have received much attention from physicists, chemists and material scientists who have been extensively investigating their optical, physical and chemical properties for fundamental understanding and technological applications (Chopra *et al.*, 2007; Lokaj *et al.*, 2001; Low *et al.*, 2002; Sathiya Moorthi *et al.*, 2005a,b; Schmalle *et al.*, 1990; Wang *et al.*, 2004). Earlier studies have indicated that chalcone and its derivatives are potential candidates for optical limiting applications (Gu *et al.*, 2008a,b). Owing to their electronic structures, chalcones also find unique applications in fluorescent probes for the sensing of metal ions (DiCesare *et al.*, 2000; Jiang *et al.*, 1994), and in biological use (Nel *et al.*, 1998). The chalcone derivatives with typical D-π-A mode have been reported to crystallize in a noncentrosymmetric crystal structure and possess second harmonic generation properties (Patil *et al.*, 2006; Patil & Dharmaprakash, 2007; Patil *et al.*, 2007*b*). In our previous investigation, the crystal structure of 1-(4-chlorophenyl)-3-(4-ethoxyphenyl)prop-2-en-1-one has been reported (Patil *et al.*, 2007*a*). To further understand the structure-property relationship, the title chalcone derivative was synthesized with ethoxy as an electron-donor group. The title compound crystallized in the non-centrosymmetric monoclinic *P*2_1_ space group and therefore it should exhibit second-order nonlinear optical properties.

The title molecule (Fig.1) is nearly planar and exists in an *E* configuration with respect to the C8═C9 double bond [1.341 (3) Å]; the C7–C8–C9–C10 torsion angle is -177.6 (2)°. The dihedral angle between rings *A* and *B* is 10.09 (11)°. The enone unit (C7–C9/O1) is essentially planar, with a maximum deviation of 0.040 (2) Å for atom C8. The mean plane through the enone unit makes dihedral angles of 12.05 (11)° and 9.87 (11)° with the planes of rings *A* and *B*, respectively. The planar ethoxy group [C13—O2—C16—C17 = 176.3 (2)°] is almost coplanar with the ring *B* [C16—O2—C13—C12 of -2.1 (3)°]. The deviations of atoms O2, C16 and C17 from ring *B* are 0.007 (2), 0.052 (3) and -0.056 (3) Å, respectively. A weak C9–H9A···O1 interaction generates an S(5) ring motif. The bond distances and angles have normal values (Allen *et al.*, 1987) and are comparable with those observed in related structures (Patil *et al.*, 2007a,b).

In the crystal structure, the molecules are linked by C—H···O hydrogen bonds (Table 1) to form a zigzag ribbon-like structure along the *b* direction (Fig.2 and Fig.3).

### Experimental

The title compound was synthesized by the condensation of 4-ethoxybenzaldehyde (0.01mol, 1.39 ml) with 3-bromoacetophenone (0.01 mol, 1.99 g)) in methanol (60 ml) in the presence of a catalytic amount of sodium hydroxide solution (5 ml, 20%). After stirring for 3 h, the contents of the flask were poured into ice-cold water (500 ml) and left to stand for 4 h. The resulting crude solid was filtered and dried. Single crystals were obtained by recrystallization from acetone.

### Refinement

All H atoms were placed in calculated positions, with C-H = 0.93 Å, *U*_iso_ = 1.2*U*_eq_(C) for aromatic and CH, C-H = 0.97 Å, *U*_iso_ = 1.2*U*_eq_(C) for CH_2_ and C-H = 0.96 Å, *U*_iso_ = 1.5*U*_eq_(C) for CH_3_ atoms. A rotating group model was used for the methyl groups. The highest residual electron density peak is located at 0.81 Å from Br1 and the deepest hole is located at 0.76 Å from Br1.

### Crystal data

**Table:**

C_17_H_15_BrO_2_	*F*_000_ = 336
*M**_r_* = 331.19	*D*_x_ = 1.572 Mg m^−^^3^
Monoclinic, *P*2_1_	Mo *K*α radiation λ = 0.71073 Å
Hall symbol: P 2yb	Cell parameters from 5989 reflections
*a* = 4.0516 (1) Å	θ = 1.1–35.0º
*b* = 9.6501 (2) Å	µ = 2.94 mm^−^^1^
*c* = 17.9120 (4) Å	*T* = 100.0 (1) K
β = 92.396 (1)º	Block, colourless
*V* = 699.72 (3) Å^3^	0.53 × 0.31 × 0.17 mm
*Z* = 2	

### Data collection

**Table:**

Bruker SMART APEXII CCD area-detector diffractometer	5989 independent reflections
Radiation source: fine-focus sealed tube	4682 reflections with *I* > 2σ(*I*)
Monochromator: graphite	*R*_int_ = 0.033
Detector resolution: 8.33 pixels mm^-1^	θ_max_ = 35.0º
*T* = 100.0(1) K	θ_min_ = 1.1º
ω scans	*h* = −6→6
Absorption correction: multi-scan(SADABS; Bruker, 2005)	*k* = −15→15
*T*_min_ = 0.305, *T*_max_ = 0.642	*l* = −28→23
14837 measured reflections	

### Refinement

**Table:**

Refinement on *F*^2^	Hydrogen site location: inferred from neighbouring sites
Least-squares matrix: full	H-atom parameters constrained
*R*[*F*^2^ > 2σ(*F*^2^)] = 0.034	*w* = 1/[σ^2^(*F*_o_^2^) + (0.0372*P*)^2^] where *P* = (*F*_o_^2^ + 2*F*_c_^2^)/3
*wR*(*F*^2^) = 0.093	(Δ/σ)_max_ = 0.002
*S* = 1.04	Δρ_max_ = 0.69 e Å^−^^3^
5989 reflections	Δρ_min_ = −0.65 e Å^−^^3^
182 parameters	Extinction correction: none
1 restraint	Absolute structure: Flack (1983), 2764 Friedel pairs
Primary atom site location: structure-invariant direct methods	Flack parameter: 0.021 (8)
Secondary atom site location: difference Fourier map	

### Special details

**Table:**

Experimental. The low-temperature data was collected with the Oxford Cyrosystem Cobra low-temperature attachment.
Geometry. All e.s.d.'s (except the e.s.d. in the dihedral angle between two l.s. planes) are estimated using the full covariance matrix. The cell e.s.d.'s are taken into account individually in the estimation of e.s.d.'s in distances, angles and torsion angles; correlations between e.s.d.'s in cell parameters are only used when they are defined by crystal symmetry. An approximate (isotropic) treatment of cell e.s.d.'s is used for estimating e.s.d.'s involving l.s. planes.
Refinement. Refinement of *F*^2^ against ALL reflections. The weighted *R*-factor *wR* and goodness of fit *S* are based on *F*^2^, conventional *R*-factors *R* are based on *F*, with *F* set to zero for negative *F*^2^. The threshold expression of *F*^2^ > σ(*F*^2^) is used only for calculating *R*-factors(gt) *etc*. and is not relevant to the choice of reflections for refinement. *R*-factors based on *F*^2^ are statistically about twice as large as those based on *F*, and *R*- factors based on ALL data will be even larger.

### Fractional atomic coordinates and isotropic or equivalent isotropic displacement parameters (Å^2^)

**Table:**

	*x*	*y*	*z*	*U*_iso_*/*U*_eq_	
Br1	0.44099 (5)	0.22151 (3)	0.509606 (11)	0.02359 (7)	
O1	0.6257 (5)	0.16393 (19)	0.80200 (10)	0.0229 (4)	
O2	0.0554 (4)	0.29630 (17)	1.23694 (9)	0.0191 (3)	
C1	0.4078 (6)	0.2599 (2)	0.66571 (13)	0.0179 (4)	
H1A	0.5320	0.1789	0.6686	0.021*	
C2	0.3124 (6)	0.3148 (2)	0.59698 (13)	0.0174 (4)	
C3	0.1291 (6)	0.4369 (2)	0.59085 (14)	0.0194 (4)	
H3A	0.0668	0.4731	0.5443	0.023*	
C4	0.0418 (6)	0.5031 (2)	0.65597 (14)	0.0194 (4)	
H4A	−0.0799	0.5848	0.6528	0.023*	
C5	0.1332 (6)	0.4494 (2)	0.72574 (14)	0.0173 (4)	
H5A	0.0725	0.4948	0.7689	0.021*	
C6	0.3170 (6)	0.3266 (2)	0.73090 (13)	0.0155 (4)	
C7	0.4277 (6)	0.2592 (2)	0.80334 (13)	0.0163 (4)	
C8	0.2936 (6)	0.3067 (2)	0.87445 (13)	0.0170 (4)	
H8A	0.1488	0.3814	0.8756	0.020*	
C9	0.3862 (5)	0.2391 (3)	0.93731 (12)	0.0162 (4)	
H9A	0.5380	0.1681	0.9313	0.019*	
C10	0.2857 (6)	0.2599 (2)	1.01363 (13)	0.0161 (4)	
C11	0.0989 (6)	0.3739 (2)	1.03642 (13)	0.0165 (4)	
H11A	0.0263	0.4389	1.0011	0.020*	
C12	0.0207 (6)	0.3914 (2)	1.11050 (13)	0.0171 (4)	
H12A	−0.0990	0.4684	1.1249	0.020*	
C13	0.1238 (6)	0.2920 (2)	1.16333 (13)	0.0156 (4)	
C14	0.3094 (6)	0.1789 (2)	1.14161 (13)	0.0169 (4)	
H14A	0.3792	0.1131	1.1768	0.020*	
C15	0.3906 (6)	0.1639 (2)	1.06790 (13)	0.0166 (4)	
H15A	0.5173	0.0884	1.0542	0.020*	
C16	−0.1265 (6)	0.4130 (3)	1.26362 (14)	0.0194 (5)	
H16A	−0.3355	0.4225	1.2356	0.023*	
H16B	−0.0010	0.4978	1.2584	0.023*	
C17	−0.1832 (9)	0.3845 (3)	1.34460 (16)	0.0323 (7)	
H17C	−0.3014	0.4605	1.3654	0.048*	
H17A	0.0256	0.3736	1.3713	0.048*	
H17B	−0.3101	0.3010	1.3488	0.048*	

### Atomic displacement parameters (Å^2^)

**Table:**

	*U*^11^	*U*^22^	*U*^33^	*U*^12^	*U*^13^	*U*^23^
Br1	0.02512 (11)	0.03165 (12)	0.01422 (9)	−0.00028 (13)	0.00341 (7)	−0.00396 (12)
O1	0.0289 (10)	0.0226 (8)	0.0170 (8)	0.0094 (7)	0.0001 (7)	0.0002 (7)
O2	0.0236 (9)	0.0196 (8)	0.0144 (8)	0.0049 (7)	0.0052 (6)	0.0037 (6)
C1	0.0184 (10)	0.0188 (10)	0.0165 (10)	−0.0003 (7)	0.0022 (8)	0.0009 (7)
C2	0.0153 (10)	0.0197 (10)	0.0173 (11)	−0.0035 (8)	0.0026 (8)	−0.0029 (8)
C3	0.0210 (11)	0.0199 (10)	0.0175 (11)	−0.0043 (9)	0.0006 (8)	0.0042 (9)
C4	0.0200 (12)	0.0149 (10)	0.0231 (12)	−0.0007 (9)	−0.0016 (9)	0.0017 (9)
C5	0.0201 (11)	0.0150 (10)	0.0166 (11)	−0.0017 (8)	0.0004 (8)	−0.0012 (8)
C6	0.0186 (10)	0.0132 (9)	0.0147 (10)	−0.0024 (8)	0.0011 (8)	−0.0004 (7)
C7	0.0179 (10)	0.0145 (9)	0.0164 (10)	−0.0011 (7)	0.0004 (8)	−0.0020 (7)
C8	0.0190 (11)	0.0168 (10)	0.0151 (10)	0.0003 (8)	0.0006 (8)	−0.0025 (8)
C9	0.0176 (9)	0.0141 (12)	0.0168 (9)	−0.0005 (8)	0.0007 (7)	−0.0023 (8)
C10	0.0172 (10)	0.0142 (9)	0.0170 (10)	−0.0027 (7)	0.0009 (8)	−0.0001 (7)
C11	0.0179 (10)	0.0150 (9)	0.0165 (10)	−0.0013 (8)	−0.0008 (8)	0.0028 (8)
C12	0.0189 (11)	0.0147 (9)	0.0176 (11)	−0.0013 (8)	0.0013 (8)	0.0003 (8)
C13	0.0144 (10)	0.0161 (10)	0.0166 (10)	−0.0015 (8)	0.0036 (8)	0.0004 (8)
C14	0.0184 (11)	0.0136 (8)	0.0186 (11)	0.0009 (7)	0.0005 (8)	0.0043 (7)
C15	0.0161 (10)	0.0150 (9)	0.0188 (11)	0.0007 (8)	0.0011 (8)	0.0004 (8)
C16	0.0232 (12)	0.0161 (10)	0.0194 (11)	−0.0015 (8)	0.0060 (9)	−0.0003 (8)
C17	0.0467 (19)	0.0282 (13)	0.0230 (14)	0.0146 (13)	0.0146 (12)	0.0044 (11)

### Geometric parameters (Å, °)

**Table:**

Br1—C2	1.897 (2)	C9—C10	1.457 (3)
O1—C7	1.221 (3)	C9—H9A	0.93
O2—C13	1.359 (3)	C10—C15	1.397 (3)
O2—C16	1.439 (3)	C10—C11	1.405 (3)
C1—C2	1.381 (3)	C11—C12	1.387 (3)
C1—C6	1.396 (3)	C11—H11A	0.93
C1—H1A	0.93	C12—C13	1.399 (3)
C2—C3	1.394 (3)	C12—H12A	0.93
C3—C4	1.389 (3)	C13—C14	1.390 (3)
C3—H3A	0.93	C14—C15	1.381 (3)
C4—C5	1.389 (4)	C14—H14A	0.93
C4—H4A	0.93	C15—H15A	0.93
C5—C6	1.401 (3)	C16—C17	1.504 (4)
C5—H5A	0.93	C16—H16A	0.97
C6—C7	1.503 (3)	C16—H16B	0.97
C7—C8	1.478 (3)	C17—H17C	0.96
C8—C9	1.341 (3)	C17—H17A	0.96
C8—H8A	0.93	C17—H17B	0.96
			
C13—O2—C16	118.31 (18)	C15—C10—C9	118.2 (2)
C2—C1—C6	119.7 (2)	C11—C10—C9	123.8 (2)
C2—C1—H1A	120.2	C12—C11—C10	121.4 (2)
C6—C1—H1A	120.2	C12—C11—H11A	119.3
C1—C2—C3	121.5 (2)	C10—C11—H11A	119.3
C1—C2—Br1	118.50 (18)	C11—C12—C13	119.3 (2)
C3—C2—Br1	119.98 (18)	C11—C12—H12A	120.3
C4—C3—C2	118.4 (2)	C13—C12—H12A	120.3
C4—C3—H3A	120.8	O2—C13—C14	115.5 (2)
C2—C3—H3A	120.8	O2—C13—C12	124.6 (2)
C3—C4—C5	121.1 (2)	C14—C13—C12	119.9 (2)
C3—C4—H4A	119.5	C15—C14—C13	120.2 (2)
C5—C4—H4A	119.5	C15—C14—H14A	119.9
C4—C5—C6	119.7 (2)	C13—C14—H14A	119.9
C4—C5—H5A	120.1	C14—C15—C10	121.2 (2)
C6—C5—H5A	120.1	C14—C15—H15A	119.4
C1—C6—C5	119.5 (2)	C10—C15—H15A	119.4
C1—C6—C7	116.3 (2)	O2—C16—C17	106.1 (2)
C5—C6—C7	124.2 (2)	O2—C16—H16A	110.5
O1—C7—C8	121.0 (2)	C17—C16—H16A	110.5
O1—C7—C6	118.8 (2)	O2—C16—H16B	110.5
C8—C7—C6	120.19 (19)	C17—C16—H16B	110.5
C9—C8—C7	118.2 (2)	H16A—C16—H16B	108.7
C9—C8—H8A	120.9	C16—C17—H17C	109.5
C7—C8—H8A	120.9	C16—C17—H17A	109.5
C8—C9—C10	129.9 (2)	H17C—C17—H17A	109.5
C8—C9—H9A	115.0	C16—C17—H17B	109.5
C10—C9—H9A	115.0	H17C—C17—H17B	109.5
C15—C10—C11	118.0 (2)	H17A—C17—H17B	109.5
			
C6—C1—C2—C3	0.7 (4)	C7—C8—C9—C10	−177.6 (2)
C6—C1—C2—Br1	179.90 (17)	C8—C9—C10—C15	172.5 (3)
C1—C2—C3—C4	−0.2 (4)	C8—C9—C10—C11	−9.9 (4)
Br1—C2—C3—C4	−179.42 (18)	C15—C10—C11—C12	0.2 (3)
C2—C3—C4—C5	−0.2 (4)	C9—C10—C11—C12	−177.5 (2)
C3—C4—C5—C6	0.1 (4)	C10—C11—C12—C13	−1.4 (4)
C2—C1—C6—C5	−0.7 (3)	C16—O2—C13—C14	178.0 (2)
C2—C1—C6—C7	179.4 (2)	C16—O2—C13—C12	−2.1 (3)
C4—C5—C6—C1	0.3 (3)	C11—C12—C13—O2	−178.4 (2)
C4—C5—C6—C7	−179.8 (2)	C11—C12—C13—C14	1.5 (3)
C1—C6—C7—O1	10.3 (3)	O2—C13—C14—C15	179.5 (2)
C5—C6—C7—O1	−169.6 (2)	C12—C13—C14—C15	−0.4 (3)
C1—C6—C7—C8	−168.7 (2)	C13—C14—C15—C10	−0.8 (4)
C5—C6—C7—C8	11.4 (3)	C11—C10—C15—C14	1.0 (3)
O1—C7—C8—C9	−2.3 (3)	C9—C10—C15—C14	178.7 (2)
C6—C7—C8—C9	176.7 (2)	C13—O2—C16—C17	176.3 (2)

### Hydrogen-bond geometry (Å, °)

**Table:**

*D*—H···*A*	*D*—H	H···*A*	*D*···*A*	*D*—H···*A*
C9—H9A···O1	0.93	2.36	2.746 (3)	105
C16—H16B···O1^i^	0.97	2.49	3.400 (3)	157

Symmetry codes:  (i) −*x*+1, *y*+1/2, −*z*+2.

## References

[bb1] Allen, F. H., Kennard, O., Watson, D. G., Brammer, L., Orpen, A. G. & Taylor, R. (1987). *J. Chem. Soc. Perkin Trans. 2*, pp. S1–S19.

[bb2] Bruker (2005). *APEX2*, *SAINT* and *SADABS* Bruker AXS Inc., Madison, Wisconsin, USA.

[bb3] Chopra, D., Mohan, T. P., Vishalakshi, B. & Guru Row, T. N. (2007). *Acta Cryst.* C**63**, o704–o710. 10.1107/S0108270107049426 18057618

[bb4] DiCesare, N. & Lakowicz, J. R. (2000). *Tetrahedron Lett.* **43**, 2615–2618. 10.1016/s0040-4039(02)00312-x PMC8022852 33828341

[bb5] Flack, H. D. (1983). *Acta Cryst.* A**39**, 876–881.

[bb6] Gu, B., Ji, W., Patil, P. S. & Dharmaprakash, S. M. (2008*a*). *J. Appl. Phys.* **103**, 103511-1–103511-6.

[bb7] Gu, B., Ji, W., Patil, P. S., Dharmaprakash, S. M. & Wang, H. T. (2008*b*). *Appl. Phys. Lett.* **92**, 091118-1–091118-3.

[bb8] Jiang, Y. B., Wang, X. J. & Lin, L. (1994). *J. Phys. Chem.* **98**, 12367–12372.

[bb9] Lokaj, J., Kettmann, V., Marchalin, S. & Sikoraiova, J. (2001). *Acta Cryst.* C**57**, 735–736. 10.1107/s0108270101003870 11408689

[bb10] Low, J. N., Cobo, J., Nogueras, M., Sánchez, A., Albornoz, A. & Abonia, R. (2002). *Acta Cryst.* C**58**, o42–o45. 10.1107/s0108270101018297 11781492

[bb11] Nel, R. J. J., Van Heerden, P. S., Van Rensburg, H. & Ferreira, D. (1998). *Tetrahedron Lett.* **39**, 5623–5626.

[bb12] Patil, P. S. & Dharmaprakash, S. M. (2007). *J. Cryst. Growth*, **3053**, 218–221.

[bb13] Patil, P. S., Fun, H.-K., Chantrapromma, S. & Dharmaprakash, S. M. (2007). *Acta Cryst.* E**63**, o2497–o2498.

[bb14] Patil, P. S., Ng, S.-L., Razak, I. A., Fun, H.-K. & Dharmaprakash, S. M. (2007). *Acta Cryst.* E**63**, o59–o60.

[bb15] Patil, P. S., Teh, J. B.-J., Fun, H.-K., Razak, I. A. & Dharmaprakash, S. M. (2006). *Acta Cryst.* E**62**, o896–o898.

[bb16] Sathiya Moorthi, S., Chinnakali, K., Nanjundan, S., Radhika, R., Fun, H.-K. & Yu, X.-L. (2005*a*). *Acta Cryst.* E**61**, o480–o482.

[bb17] Sathiya Moorthi, S., Chinnakali, K., Nanjundan, S., Selvam, P., Fun, H.-K. & Yu, X.-L. (2005*b*). *Acta Cryst.* E**61**, o743–o745.

[bb18] Schmalle, H. W., Adiwidjaja, G., Jarchow, O. H., Hausen, B. M. & Wollenweber, E. (1990). *Acta Cryst.* C**46**, 1712–1715. 10.1107/s0108270190000221 2088422

[bb19] Sheldrick, G. M. (2008). *Acta Cryst.* A**64**, 112–122. 10.1107/S0108767307043930 18156677

[bb20] Spek, A. L. (2003). *J. Appl. Cryst.* **36**, 7–13.

[bb21] Wang, L., Zhang, Y., Lu, C.-R. & Zhang, D.-C. (2004). *Acta Cryst.* C**60**, o696–o698. 10.1107/S0108270104017883 15345860

